# A Hybrid AI-Assisted Framework for Anxiety Support: System Description and Exploratory Findings Among University Students

**DOI:** 10.3390/bs16101781

**Published:** 2026-09-30

**Authors:** Parastou Rostami, Elaheh Bakhtiari, Benyamin Baharizadeh, Giorgio M. Grasso

**Affiliations:** NISC Lab, Cognitive Science Department, University of Messina, 98121 Messina, Italy; ebakhtiari@unime.it (E.B.); benyamin.baharizadeh@studenti.unime.it (B.B.); giorgiomario.grasso@unime.it (G.M.G.)

**Keywords:** anxiety, digital mental health, embodied conversational avatar, AI-assisted mental health support, hybrid care

## Abstract

A growing number of individuals experiencing anxiety and other mental health difficulties require accessible approaches to mental health support. This paper presents a hybrid AI-assisted framework designed as a potential complementary support tool for future therapist-supervised mental health care rather than as a replacement for clinical decision-making. The framework integrates a flexible dialogue-generation architecture, an interactive embodied avatar, and an ethical and safety-oriented component emphasizing privacy and human-centered governance. An exploratory survey including structured and open-ended questions was conducted with 102 university students following a live demonstration of the proposed framework. Overall, 76.2% of participants agreed or strongly agreed that AI could help some people with mental health support, 59.4% indicated that they would use AI-assisted support if freely available, while 55.0% preferred interacting with a human therapist. Open-ended responses highlighted accessibility and continuous availability as potential advantages, while concerns included emotional understanding, privacy, and the accuracy and reliability of AI-generated responses. These findings provide preliminary information about university students’ perceptions following exposure to the proposed framework but do not constitute an evaluation of therapeutic effectiveness, clinical utility, or clinical safety. Further research involving direct user interaction, clinically relevant populations, validated outcome measures, and appropriate safety protocols is needed.

## 1. Introduction

Mental health disorders, particularly anxiety-related conditions, represent major public health challenges and can substantially affect emotional well-being, academic and occupational functioning, and interpersonal relationships (Patel et al., 2018). Despite increasing demand for psychological support, access to timely mental health care remains limited by factors including shortages of mental health professionals, financial barriers, stigma, and long waiting times (Patel et al., 2018; Rowan et al., 2013). These challenges have contributed to growing interest in accessible digital approaches to mental health support.

Recent developments in artificial intelligence (AI), large language models (LLMs), and conversational systems have expanded the possibilities for digital mental health support (Adindu et al., 2025; Zhang et al., 2025). Conversational AI systems may provide accessible and potentially personalized forms of interaction, while embodied conversational avatars can additionally incorporate verbal and nonverbal features such as facial expressions, gaze behavior, gesture animation, and speech into digital interaction (Provoost et al., 2017; Rehm et al., 2016; Pinto et al., 2025). These characteristics may influence perceptions of social presence and interpersonal interaction, although richer interaction does not by itself establish therapeutic effectiveness or clinical safety.

At the same time, the use of AI in mental health contexts raises important scientific, ethical, and safety considerations. These include limitations in contextual and emotional understanding, uncertainty regarding the accuracy and reliability of AI-generated responses, privacy and data-protection concerns, accountability, and the management of potentially high-risk situations (Palmer & Schwan, 2025; Zhang et al., 2025). These considerations have increased interest in hybrid human–AI approaches in which AI-based systems are positioned as complementary tools within models that retain an important role for human professionals (Kiuchi et al., 2024; Palmer & Schwan, 2025).

Despite these developments, important gaps remain in the current literature. Although previous studies have examined text-based conversational agents and embodied virtual avatars, comparatively less attention has been given to integrating embodied conversational interaction, adaptive dialogue generation, and clinician-informed prompting within a unified framework. In addition, the perceptions of university students following exposure to such hybrid AI-assisted mental health systems remain insufficiently explored.

Building on these considerations, the present study proposes a hybrid AI-assisted framework for anxiety-related mental health support that integrates an embodied conversational avatar, multimodal interaction, adaptive dialogue generation, and clinician-informed prompting strategies. The framework is positioned as a potential complementary support tool for future therapist-supervised mental health care rather than as an autonomous therapeutic or clinical decision-making system.

The primary aim of the study was to present the proposed framework and explore university students’ perceptions and attitudes toward AI-assisted mental health support following exposure to a live demonstration. The empirical component focused on perceived usefulness, accessibility-related perceptions, emotional understanding, privacy concerns, and willingness to consider AI-assisted support.

Research Question: How do university students perceive the proposed hybrid AI-assisted mental health support framework following exposure to a live demonstration, and what strengths, concerns, and potential barriers do they identify regarding its use as a complementary support tool?

The present study was not designed to evaluate therapeutic effectiveness, anxiety symptom reduction, clinical safety, therapeutic alliance, or other clinical outcomes.

### 1.1. AI-Assisted Mental Health Systems

AI-assisted mental health technologies have evolved from earlier rule-based conversational agents toward more advanced systems that can support context-sensitive and adaptive forms of interaction (Adindu et al., 2025; Zhang et al., 2025). Previous research suggests that AI-based conversational systems may support mental health-related outcomes, including reductions in psychological distress and depressive symptoms, while also offering opportunities for accessible and personalized digital support (Li et al., 2023; Zhang et al., 2025; Adindu et al., 2025).

Evidence from systematic reviews and meta-analyses further suggests that conversational agent interventions may produce short-term improvements across several mental health outcomes. A meta-analysis of 32 randomized controlled trials involving 6089 participants found statistically significant short-term effects on depressive symptoms, generalized and specific anxiety symptoms, psychological distress, stress, and related outcomes, although these effects were not consistently maintained over longer follow-up periods (He et al., 2023).

Similarly, a systematic review and meta-analysis of 35 studies, including 15 randomized controlled trials, found significant effects of AI-based conversational agents on depressive symptoms and psychological distress, while effects on overall psychological well-being were not statistically significant (Li et al., 2023).

These findings provide empirical support for the potential of conversational AI in mental health contexts while also highlighting heterogeneity across interventions, populations, outcomes, and study designs. Embodied virtual avatars have been investigated as a means of incorporating verbal and nonverbal interaction into digital mental health systems and may contribute to user engagement and perceptions of interpersonal interaction (Provoost et al., 2017; Rehm et al., 2016; Pinto et al., 2025).

#### Embodied Conversational Avatars and the Proposed Framework

Although previous research has examined conversational AI, virtual agents, and embodied avatars in digital mental health, fewer studies have explicitly examined hybrid frameworks that combine embodied conversational interaction with clinician-informed therapeutic principles while positioning AI as a complementary rather than autonomous support tool. Existing evidence suggests that the characteristics of conversational agents may influence their psychological and interactional effects. For example, a systematic review and meta-analysis of AI-based conversational agents reported that multimodal or voice-based systems showed somewhat greater effects on psychological distress than text-based systems, while user experience was associated with factors such as the human–AI therapeutic relationship, content engagement, and communication quality (Li et al., 2023). In addition, systematic evidence on digital psychotherapy indicates that therapeutic alliance can be established through computer-mediated communication and is generally associated with treatment outcomes, although important relational dimensions may not be fully captured by existing measures (van Lotringen et al., 2021). These findings provide a rationale for incorporating embodied and multimodal interaction into AI-assisted mental health systems while recognizing that richer interaction does not, by itself, establish therapeutic effectiveness or clinical safety.

**Figure 1:**
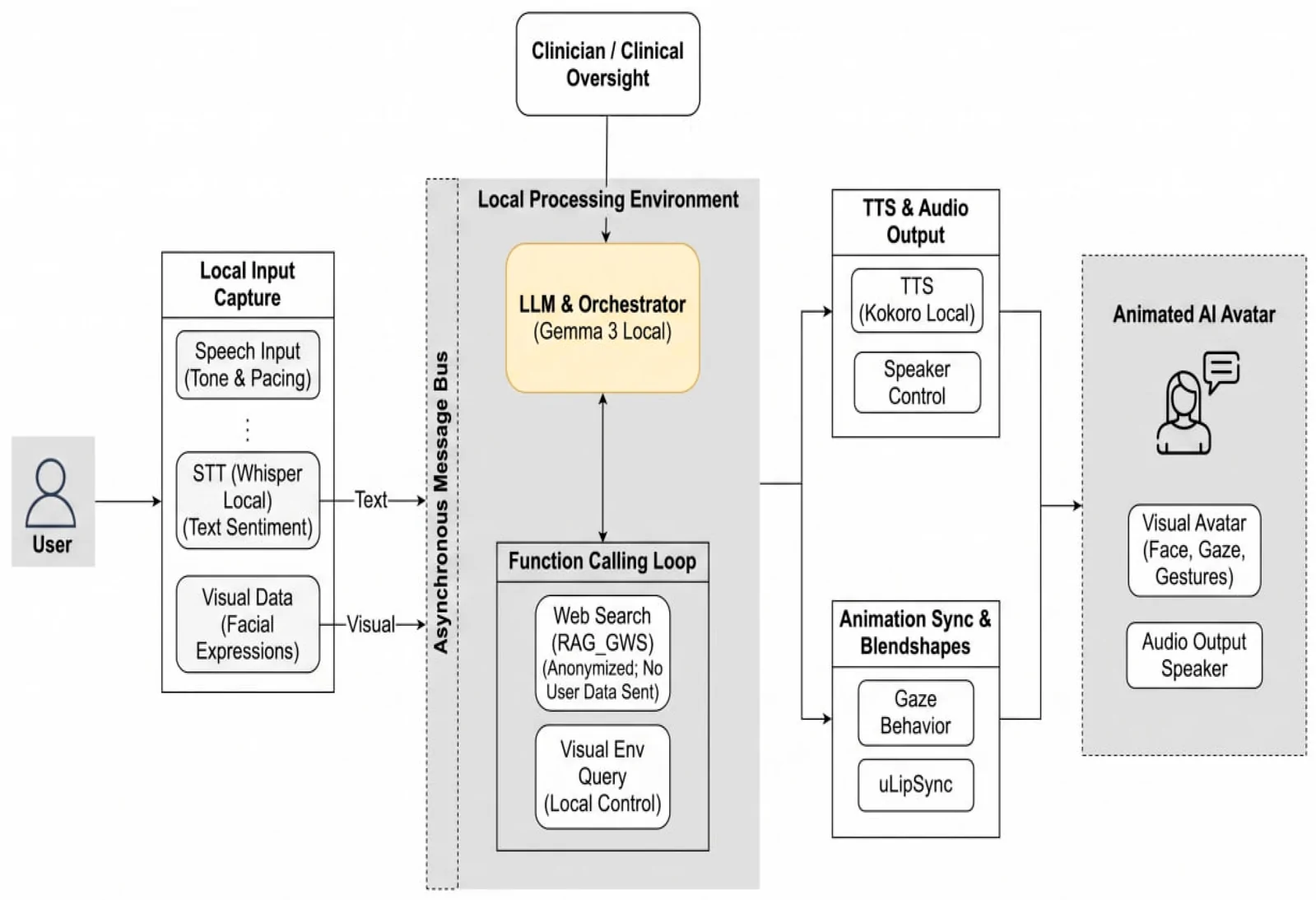
Avatar’s system diagram. This block diagram shows the complete technical infrastructure of the project, as it is used in experiments.. Figure 1 provides an overview of the system architecture, including the user interface, speech-processing components, local LLM inference, speech synthesis, avatar rendering, and clinician-informed conversational constraints. Components that were supported by the broader implementation but were not used during participants’ exposure to the system are identified separately.

Building on this literature, the present framework integrates an embodied conversational avatar with multimodal interaction, adaptive dialogue generation, and clinician-informed prompting strategies. The use of clinician-informed prompting is intended to incorporate psychologically informed communication principles and predefined conversational constraints while maintaining the AI system in a complementary support role. The framework is therefore positioned as a potential complementary support tool rather than an autonomous therapeutic system. An overview of the proposed system architecture and its main components is presented in Figure 1. The empirical component of the study subsequently provides a preliminary exploratory assessment of university students’ perceptions following exposure to a live demonstration of the proposed approach.

## 2. Methodology

### 2.1. Study Design

The present study employed an exploratory survey design to assess university students’ perceptions and attitudes toward AI-assisted mental health support following exposure to a demonstration of the proposed framework. The proposed framework was designed for potential future application in anxiety-related mental health support; however, the empirical component of the present study did not assess anxiety symptoms, anxiety diagnoses, or anxiety-specific clinical outcomes. Instead, the study focused on participants’ attitudes toward AI-assisted mental health support, perceived usefulness and accessibility, perceptions of the system’s emotional understanding, privacy concerns, and willingness to engage with such systems.

### 2.2. Participants

A total of 102 university students voluntarily participated in the study. Participants were recruited through convenience sampling using an online Google Forms questionnaire distributed among university students at the University of Messina, Italy, between March and April 2026. Approximately 200 students were invited to participate, corresponding to an approximate response rate of 51%. No a priori sample-size or statistical power calculation was performed because the study was designed as a preliminary exploratory assessment of participants’ perceptions rather than as a hypothesis-testing clinical study.

Eligibility was limited to individuals currently enrolled at a university who agreed to participate. Questionnaires that were incomplete at the overall survey level were excluded from the analysis. All 102 participants provided sufficient responses to be included in the final sample, although some individual questionnaire items had missing responses. Therefore, item-level denominators varied across analyses, and missing responses were excluded from the denominator for the corresponding item. No financial or other compensation was provided.

University students were selected as an accessible population for this exploratory assessment of general perceptions of digital and AI-assisted mental health support. Anxiety symptoms, anxiety diagnoses, current psychological distress, and treatment needs were not assessed. Therefore, the sample should not be considered representative of individuals experiencing anxiety or seeking psychological treatment.

The sample characteristics are presented in Table 1. Regarding previous psychological support or psychotherapy experience, 36 participants (35.3%) reported no previous experience, 48 (47.1%) reported previous experience, and 18 (17.6%) reported current experience.

#### 2.2.1. Proposed AI-Assisted Framework

The proposed AI-assisted framework consisted of a three-dimensional conversational avatar developed within an interactive virtual environment. The avatar was implemented in Unity 6 using the Genesis 9 character model (Unity Technologies, n.d.; Daz 3D, 2026). The framework incorporated a multimodal computational pipeline combining local speech recognition, large language model–based response generation, adaptive conversational processing, and local speech synthesis. Local speech recognition was implemented using Whisper (Radford et al., 2023), while the overall architecture was designed to generate context-sensitive conversational responses based on user input.

The system was not equipped with an independent facial-expression, physiological, or other validated affect-recognition model for the clinical assessment of users’ emotional states. Accordingly, references to affective adaptation in the present manuscript refer to context-sensitive conversational response generation based on the interaction context rather than to validated clinical emotion, anxiety, or psychological-state assessment.

The framework also incorporated a function-calling mechanism to facilitate interaction with the virtual environment and access to supplementary information. The Visual Environment Query operated under local control and was designed to assess the user’s immediate physical context within the virtual environment. When necessary, a Web Search function (RAG_GWS) was used to retrieve general psychoeducational information. Retrieval queries were designed to minimize the inclusion of sensitive user data or personal health information. However, the present study did not include a formal privacy, data-protection, or cybersecurity assessment; therefore, the privacy-related characteristics of the framework should not be interpreted as constituting a formal security evaluation.

**Figure 2:**
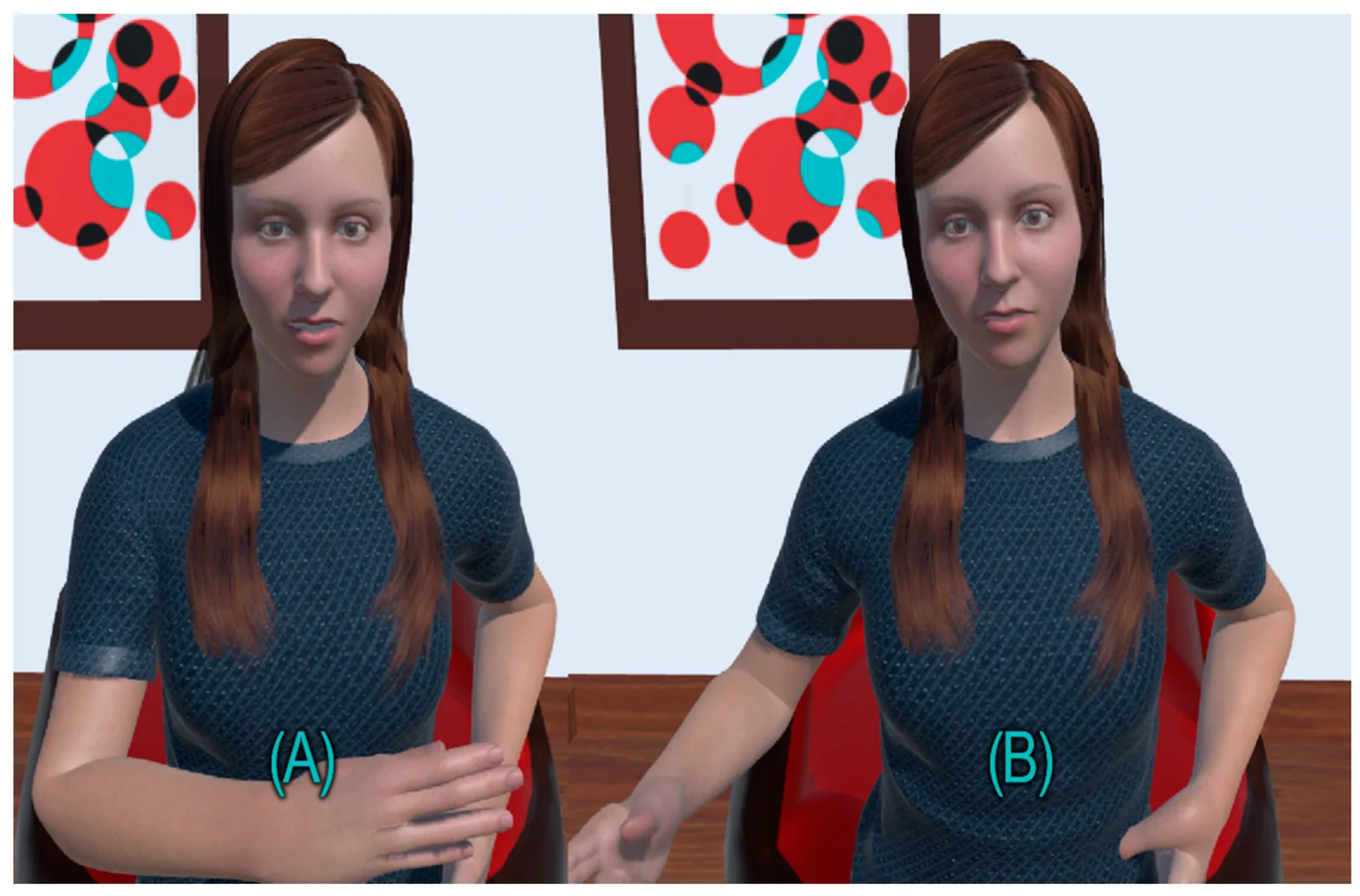
Embodied conversational avatar design. (**A**) Avatar with a hand gesture positioned in front of the body. (**B**) Avatar with an open-hand gesture.

The avatar was designed to support natural conversational interaction through synchronized facial expressions, gaze behavior, lip synchronization, and adaptive verbal responses. Examples of the embodied conversational avatar design and gesture configurations are shown in Figure 2. Clinician-informed principles were incorporated at the framework-design level through CBT-informed prompting strategies and predefined conversational constraints. These design elements were intended to promote psychologically informed, nonjudgmental, and context-sensitive communication while maintaining the AI system in a complementary support role rather than positioning it as an autonomous therapeutic or clinical decision-making system.

Importantly, no clinician continuously monitored the system, reviewed AI-generated responses, or supervised participants’ exposure to the system during the present study. Therefore, the term “clinical oversight,” where used in the manuscript, refers to clinician-informed system design and prompting rather than real-time clinical supervision. The framework was consequently developed as a potential complementary support system for future human–AI mental health applications, while its therapeutic effectiveness, clinical safety, and suitability for clinical populations remain to be established through future research.

#### 2.2.2. Measures and Questionnaire

A structured 24-item questionnaire was developed specifically for this exploratory study to assess participants’ general perceptions, attitudes, and willingness regarding AI-assisted mental health support. The questionnaire was informed by previous literature on digital mental health, conversational agents, embodied conversational agents, and AI-assisted mental health support, including research addressing user perceptions, accessibility, interaction, and concerns related to AI-mediated psychological support (Provoost et al., 2017; Rehm et al., 2016; Adindu et al., 2025; Zhang et al., 2025). The items were developed to address aspects of AI-assisted mental health support relevant to the exploratory aims of the study. The questionnaire was not adapted from a single previously validated psychometric instrument and was therefore treated as a study-specific exploratory questionnaire rather than a validated measurement scale.

The questionnaire comprised 24 items across six sections: (1) informed consent (1 item); (2) demographic and background information (6 items); (3) willingness to interact with AI (7 items); (4) opinions about artificial intelligence (7 items); (5) open-ended questions (2 items); and (6) general willingness to participate in this type of research (1 item). The questionnaire included demographic and multiple-choice questions, five-point Likert-scale items, and open-ended questions.

The Likert-scale items used five response options. For agreement-based items, responses ranged from 1 = strongly disagree to 5 = strongly agree. For other items, response anchors were specified according to the aspect being assessed (e.g., 1 = not at all comfortable to 5 = very comfortable; 1 = not at all useful to 5 = very useful). The questionnaire addressed individual aspects of participants’ perceptions, including comfort with AI interaction, perceived emotional understanding, preference for human versus AI support, perceived usefulness, privacy-related concerns, willingness to use AI-assisted support if freely available, and perceived potential benefit of AI for some individuals. The seven items presented in Table 2 were drawn from the opinions-about-AI section of the questionnaire and were analyzed individually. These items were not treated as a validated multi-item scale, and no composite score was calculated. Accordingly, terms such as perceived emotional understanding, perceived usefulness, and willingness refer to the content of individual questionnaire items and should not be interpreted as established psychometric constructs.

The questionnaire was reviewed by the research team for clarity and comprehensibility before administration. No formal pilot administration or pilot testing was conducted before the main study. The questionnaire was developed specifically for the exploratory aims of the present study and was therefore treated as a study-specific exploratory questionnaire rather than a validated measurement instrument. The complete questionnaire, including participant instructions and response options, is provided in Supplementary Material.

The questionnaire also included two open-ended questions addressing participants’ main concerns about interacting with an AI chat and their motivations for participating in an AI-related research study. A total of 85 participants provided responses to the concerns question, while 80 participants provided responses to the motivation question. The responses were reviewed descriptively by the research team to identify recurring topics and patterns. No formal qualitative coding framework, inferential qualitative analysis, independent coding, or inter-rater reliability assessment was performed. The open-ended responses were used to complement the structured survey findings and were not treated as psychometric measures.

### 2.3. Procedure

Participants completed a structured evaluation procedure consisting of four stages: (1) introduction and orientation to the proposed AI-assisted mental health support framework; (2) presentation of the embodied conversational avatar and its intended objectives; (3) a live demonstration of the proposed system conducted by the researcher; and (4) completion of the study questionnaire assessing participants’ general perceptions and attitudes toward AI-assisted mental health support, including perceived usefulness, willingness to use AI-assisted support, perceived emotional understanding, preference for human versus AI-based support, and concerns regarding the use of AI in mental health contexts.

The questionnaire was administered online using Google Forms and was presented in English. Informed consent was presented within the Google Forms questionnaire before participants proceeded to the study questions. In addition, a separate written informed consent form was completed before participation in the study. The questionnaire was administered to international university students. As international students were required to demonstrate a minimum IELTS score of 6 for university admission, participants had demonstrated English-language proficiency considered sufficient for university study and completion of the English-language questionnaire.

Participants did not directly interact with the AI avatar during the study. Instead, all participants observed a live demonstration of the proposed system conducted by the researcher. Demonstrations were conducted either individually or in small groups, depending on the session, and lasted less than 10 min. No standardized script or predefined conversational scenario was used. Consequently, the specific conversational content and prompts could vary naturally across sessions. However, the same AI avatar system, principal system features and objectives, and overall procedural sequence were maintained across sessions. The general sequence consisted of the introduction, system presentation, live demonstration, and subsequent questionnaire completion.

The entire evaluation procedure took approximately 10 min per participant, including the system demonstration and questionnaire completion. Accordingly, the present procedure represents an exploratory assessment of participants’ perceptions following exposure to a demonstration of the proposed framework. It should not be interpreted as a direct evaluation of usability, feasibility, therapeutic effectiveness, or clinical safety.

### 2.4. Statistical Analysis

Descriptive statistical analyses were performed using Microsoft Excel. Frequencies and percentages were calculated to summarize categorical and Likert-scale responses, while means and standard deviations were calculated where appropriate. For the individual five-point Likert-scale items, medians and interquartile ranges (IQRs) were also calculated to describe the central tendency and dispersion of responses. Percentages were calculated based on the number of valid responses for each individual item. Missing responses were excluded from the denominator for the corresponding item and were not imputed.

Exploratory subgroup comparisons were conducted to examine whether responses differed according to gender and previous experience with psychological support or psychotherapy. Because the questionnaire items were ordinal five-point Likert-type responses, the Mann–Whitney U test was used to compare female and male participants, while the Kruskal–Wallis test was used to compare three groups according to psychological support or psychotherapy experience: no previous experience, previous experience, and current experience. Inferential analyses were performed using Python version 3.13.5 with SciPy version 1.17.0. All tests were two-sided, and statistical significance was defined as *p* < 0.05. No adjustment for multiple testing was applied because the subgroup analyses were exploratory. Accordingly, the resulting *p*-values were interpreted cautiously and were not considered confirmatory evidence.

For the Mann–Whitney U analyses, rank-biserial correlation was used as an effect-size measure. For the Kruskal–Wallis analyses, epsilon-squared (ε^2^) was used as an estimate of effect size and was calculated as: ε^2^ = (H − k + 1)/(N − k), where H is the Kruskal–Wallis test statistic, k is the number of groups, and N is the total number of valid observations included in the analysis. Negative epsilon-squared estimates were interpreted as negligible effects and reported as 0. The single non-binary participant was not included in the gender comparison because a meaningful inferential comparison could not be performed for this subgroup. Given the exploratory nature of these analyses, effect sizes were interpreted cautiously.

Confidence intervals were not calculated because the primary purpose of the study was to provide descriptive and exploratory information about participants’ perceptions rather than population-level estimates or confirmatory statistical inference.

Participants’ fields of study were recorded and summarized descriptively in Table 1 but were not included in inferential subgroup analyses because the distribution across academic fields was uneven, with some categories containing relatively few participants, including only four participants in the business/management category. Similarly, prior chatbot use, technical familiarity with AI, and general attitudes toward technology were not included in inferential analyses because these characteristics were not systematically assessed in the questionnaire. Mental health status, anxiety symptoms, current psychological distress, and treatment needs were not included in the analyses because these variables were not assessed in the study.

The two open-ended questions were summarized descriptively by reviewing recurring topics and patterns in participants’ responses. No formal qualitative coding framework, inferential qualitative analysis, independent coding, or inter-rater reliability assessment was performed.

### 2.5. Ethics

The study was approved by the Ethics Committee of the COSPECS Department, University of Messina (Approval Code: COSPECS_12_2026; Approval Date: 2 March 2026). All participants provided informed consent before participation in the study. Informed consent was presented within the Google Forms questionnaire, and a separate written informed consent form was also completed before participation.

## 3. Results

### 3.1. Overall User Perceptions

Table 2 presents participants’ responses to the seven individual Likert-scale items assessing perceptions of AI-assisted mental health support. Median responses ranged from 3 to 4, with an IQR of 1 for all seven items. Percentages were calculated based on valid responses for each individual item.

Among valid responses, 58.4% of participants agreed or strongly agreed that they would feel comfortable talking about their problems with an AI-powered therapist, while 59.0% agreed or strongly agreed that an AI-powered therapist could be useful for psychological support. In addition, 76.2% agreed or strongly agreed that an AI-powered therapist could help some people, and 59.4% indicated that they would be willing to use an AI-powered therapist if it were free.

In contrast, 23.8% of participants agreed or strongly agreed that an AI-powered therapist could understand their emotions, while 55.0% agreed or strongly agreed that they would prefer talking to a real person rather than an AI-powered therapist. Regarding information sharing, 23.8% agreed or strongly agreed that they would feel uncomfortable sharing information with AI.

These findings describe participants’ perceptions across individual questionnaire items. They should not be interpreted as evidence of therapeutic effectiveness, clinical usefulness, or validated psychological constructs, as the questionnaire was developed specifically for this exploratory study and the items were analyzed individually.

### 3.2. Exploratory Subgroup Comparisons

Exploratory subgroup analyses were conducted to examine whether responses to the seven individual attitude items differed according to gender and previous experience with psychological support or psychotherapy.

For gender, responses from female participants (n = 52) and male participants (n = 49) were compared using the Mann–Whitney U test. The single non-binary participant was not included in the inferential gender comparison because the subgroup size did not permit a meaningful statistical comparison. No statistically significant differences were observed between female and male participants for any of the seven questionnaire items. The Mann–Whitney U statistics, *p* values, and rank-biserial correlations were as follows: comfort talking about problems with an AI-powered therapist, U = 1416.5, *p* = 0.249, rrb = 0.135; perceived ability of an AI-powered therapist to understand emotions, U = 1240.0, *p* = 0.959, rrb = −0.006; preference for talking to a real person rather than an AI-powered therapist, U = 1303.5, *p* = 0.574, rrb = 0.067; perceived usefulness of an AI-powered therapist for psychological support, U = 1407.5, *p* = 0.197, rrb = 0.152; discomfort with sharing personal information with an AI-powered therapist, U = 1227.0, *p* = 0.888, rrb = −0.017; willingness to use an AI-powered therapist if free, U = 1396.0, *p* = 0.310, rrb = 0.119; and perceived ability of an AI-powered therapist to help some people, U = 1379.0, *p* = 0.370, rrb = 0.105.

Previous or current experience with psychological support or psychotherapy was reported by 66 participants (64.7%). Responses were therefore compared across three groups: no previous experience (n = 36), previous experience (n = 48), and current experience (n = 18), using the Kruskal–Wallis test. No statistically significant differences were observed across the three groups for any of the seven questionnaire items. The Kruskal–Wallis H statistics, *p* values, and epsilon-squared (ε^2^) effect-size estimates were as follows: comfort talking about problems with an AI-powered therapist, H = 0.756, *p* = 0.685, ε^2^ = 0; perceived ability of an AI-powered therapist to understand emotions, H = 1.439, *p* = 0.487, ε^2^ = 0; preference for talking to a real person rather than an AI-powered therapist, H = 1.701, *p* = 0.427, ε^2^ = 0; perceived usefulness of an AI-powered therapist for psychological support, H = 0.815, *p* = 0.665, ε^2^ = 0; discomfort with sharing personal information with an AI-powered therapist, H = 5.675, *p* = 0.059, ε^2^ = 0.037; willingness to use an AI-powered therapist if free, H = 2.241, *p* = 0.326, ε^2^ = 0.002; and perceived ability of an AI-powered therapist to help some people, H = 1.285, *p* = 0.526, ε^2^ = 0. The item concerning discomfort with sharing personal information with an AI-powered therapist was not statistically significant (*p* = 0.059).

Overall, the exploratory subgroup analyses did not identify statistically significant differences in responses to the seven questionnaire items according to gender or previous psychological support or psychotherapy experience.

### 3.3. Open-Ended Responses

The open-ended responses provided additional descriptive information regarding participants’ perceptions and concerns about AI-assisted mental health support. A total of 85 participants provided responses to the question concerning their main concerns about interacting with an AI chat, while 80 participants provided responses regarding their motivations for participating in an AI-related research study.

Among the recurring concerns reported by participants were limited emotional understanding and empathy, uncertainty regarding the accuracy and reliability of AI-generated responses, and privacy and data-security concerns related to sharing personal information with an AI system. Some participants also expressed a preference for human interaction and concerns about the limitations of AI in understanding personal or emotional experiences.

Regarding motivations for participating in an AI-related research study, participants mentioned accessibility and continuous availability as potential advantages, including the possibility of accessing support without waiting for an appointment.

The open-ended responses were reviewed descriptively by the research team to identify recurring topics and patterns. No formal qualitative coding framework, inferential qualitative analysis, independent coding, or inter-rater reliability assessment was performed. The recurring topics reported in this section were not predefined response options; rather, they were identified descriptively from participants’ open-ended responses. These responses were used to complement the findings from the structured questionnaire items.

### 3.4. Summary of Findings

Overall, the structured survey responses showed that a proportion of participants reported favorable attitudes toward AI-assisted mental health support. Specifically, 59.0% of participants agreed or strongly agreed that an AI-powered therapist could be useful for psychological support, 76.2% agreed or strongly agreed that AI could help some people, and 59.4% indicated that they would be willing to use AI-assisted support if it were freely available.

At the same time, participants reported several reservations. Only 23.8% agreed or strongly agreed that an AI-powered therapist could understand their emotions, while 55.0% agreed or strongly agreed that they would prefer talking to a real person rather than an AI-powered therapist. In addition, 23.8% agreed or strongly agreed that they would feel uncomfortable sharing personal information with an AI-powered therapist. Recurring topics identified descriptively in the open-ended responses included concerns regarding emotional understanding and empathy, privacy and data security, and the accuracy and reliability of AI-generated responses.

Taken together, these findings describe participants’ reported attitudes and perceptions following exposure to a demonstration of the proposed framework. They provide preliminary information about perceptions of AI-assisted mental health support within the participating university-student sample but do not provide evidence regarding therapeutic effectiveness, anxiety reduction, clinical utility, or clinical safety.

## 4. Discussion

The present study explored university students’ perceptions and attitudes toward AI-assisted mental health support following exposure to a live demonstration of the proposed framework. Overall, the findings showed variation across the individual questionnaire items. A proportion of participants perceived AI-assisted support as potentially useful and accessible, while reservations remained regarding emotional understanding, privacy, reliability, and preference for human interaction. Importantly, these findings reflect participants’ perceptions following exposure to a demonstration of the proposed framework and do not constitute evidence of therapeutic effectiveness, anxiety reduction, clinical utility, or clinical safety.

The finding that 59.0% of participants agreed or strongly agreed that an AI-powered therapist could be useful for psychological support suggests that a substantial proportion of the participating students recognized a potential supportive role for AI in mental health contexts. Similarly, 76.2% agreed or strongly agreed that an AI-powered therapist could help some people, and 59.4% indicated that they would be willing to use such support if it were freely available. These responses may reflect perceived advantages associated with accessibility and availability, particularly because AI-based systems may potentially be accessed without the scheduling constraints associated with traditional face-to-face services. This interpretation is broadly consistent with previous literature suggesting that AI-assisted and conversational mental health technologies may offer opportunities for accessible and personalized forms of support (Ayoola et al., 2025; Zhang et al., 2025). Systematic reviews and meta-analyses have also reported potential short-term benefits of conversational agents across several mental health outcomes, including depressive symptoms, psychological distress, and anxiety-related outcomes (He et al., 2023; Li et al., 2023). However, these findings concern interventions or conversational systems evaluated in other studies, whereas the present study examined perceptions following a brief demonstration. The present findings therefore indicate perceived potential rather than demonstrated therapeutic benefit.

The pattern of responses also suggests that perceived usefulness and willingness to use AI-assisted support did not necessarily imply confidence in the emotional capabilities of such systems. Only 23.8% of participants agreed or strongly agreed that an AI-powered therapist could understand their emotions, while 55.0% indicated a preference for talking to a real person rather than an AI-powered therapist. This distinction is important because mental health support involves not only the delivery of information or conversational responses but also interpersonal processes such as empathy, trust, emotional attunement, and the perception of being understood. The open-ended responses similarly identified limited emotional understanding and empathy as recurring concerns. Previous literature has emphasized that trust, relational qualities, privacy, and perceptions of the limitations of AI-mediated support may influence how users evaluate AI-based mental health technologies (Békés & Aafjes-van Doorn, 2026; Palmer & Schwan, 2025; Zhang et al., 2025). The present findings are therefore consistent with a broader pattern in which users may recognize the potential practical value of AI while remaining cautious about its capacity to reproduce important interpersonal aspects of human psychological support.

Privacy and reliability concerns provide a further explanation for this cautious pattern. Participants identified uncertainty regarding the accuracy and reliability of AI-generated responses, as well as concerns about privacy and data security, among the recurring topics in the open-ended responses. These concerns are particularly relevant in mental health contexts because users may disclose highly personal or sensitive information when seeking support. Even when an AI system is designed to process information locally or to minimize the transmission of sensitive information, users’ perceptions of privacy and trust remain important determinants of their willingness to engage with such systems. The present study did not formally evaluate privacy, cybersecurity, or the factual reliability of AI-generated responses; therefore, these findings should be interpreted as participants’ concerns rather than as evidence of specific technical deficiencies in the proposed framework.

Taken together, the findings support positioning AI-assisted mental health systems as complementary tools rather than replacements for human professionals. The proposed framework incorporates clinician-informed principles, CBT-informed prompting strategies, and predefined conversational constraints, but these design features should not be interpreted as equivalent to clinical supervision. The present study did not involve continuous clinical supervision, clinician review of AI-generated responses, or direct clinical oversight during participant exposure to the system. Accordingly, the clinician-informed component of the framework should be understood as a design principle intended to promote psychologically informed and context-sensitive communication rather than as evidence of demonstrated clinical safety. Future research should investigate how human oversight can be incorporated into AI-assisted mental health systems and how responsibility can be appropriately distributed between AI systems and qualified professionals.

The use of embodied conversational avatars may further influence users’ expectations and perceptions. Previous research has examined virtual agents and embodied conversational systems as a means of incorporating verbal and nonverbal interaction into digital mental health applications (Pinto et al., 2025; Provoost et al., 2017; Rehm et al., 2016). Other research has explored adaptive conversational approaches informed by psychologically relevant principles, including CBT-oriented conversational strategies (Shidara et al., 2024). The combination of embodied interaction, adaptive dialogue generation, and clinician-informed design therefore provides a plausible basis for further investigation. At the same time, embodiment may increase the extent to which users attribute human-like characteristics or emotional capabilities to an AI system. This possibility makes it particularly important to distinguish naturalistic interaction from actual emotional understanding or clinical competence. The present study did not directly evaluate these processes, and future research should examine whether embodied features influence trust, perceived empathy, expectations, engagement, and appropriate understanding of the system’s limitations.

The findings also have implications for the ethical and responsible development of AI-assisted mental health systems. Important considerations include algorithmic bias, accountability for AI-generated responses, informed consent, privacy and data protection, management of high-risk or crisis situations, anthropomorphic perceptions, and the possibility of excessive reliance on AI-based support (Adindu et al., 2025; Békés & Aafjes-van Doorn, 2026; Kiuchi et al., 2024; Palmer & Schwan, 2025). Informed consent is particularly important because users should understand that interaction with an AI system differs from interaction with a qualified mental health professional. Similarly, appropriate human oversight and accountability mechanisms are necessary when AI-generated responses may influence users’ perceptions or decisions in mental health contexts. These considerations were incorporated into the broader design rationale of the proposed framework but were not empirically evaluated in the present study.

### Implications for Future Research and Framework Development

The findings have several implications for future research and the development of AI-assisted mental health frameworks. First, the difference between perceived usefulness and perceived emotional understanding suggests that future evaluations should consider multiple dimensions of user experience rather than relying solely on willingness to use or perceived accessibility. Direct assessment of trust, perceived empathy, emotional understanding, communication quality, and therapeutic alliance may help clarify which characteristics of embodied and conversational AI are most relevant to users’ experiences.

Second, future studies should move beyond demonstration-based evaluation and examine direct interaction with the proposed framework. Studies involving standardized interaction procedures and appropriate comparison conditions could help determine whether the combination of embodied interaction, adaptive dialogue generation, and clinician-informed prompting provides measurable benefits compared with other forms of digital mental health support.

Third, future research should combine measures of user perceptions with validated psychological and clinical outcome measures. This would allow researchers to distinguish perceived usefulness and attitudes toward AI-assisted support from changes in psychological symptoms or other clinically relevant outcomes. Research involving clinically relevant populations, longitudinal follow-up, and adequately powered samples would also be needed before conclusions could be drawn regarding potential clinical applications.

Finally, future development should incorporate clearly defined human-oversight and safety mechanisms. These should include procedures for managing high-risk or crisis-related situations, monitoring potentially inappropriate or harmful AI-generated responses, protecting sensitive information, and defining the responsibilities of AI systems and qualified mental health professionals. Such considerations are particularly important when AI-assisted systems are designed for contexts involving psychologically vulnerable users.

## 5. Limitations

Several limitations should be considered when interpreting the findings of this study.

First, the relatively small sample size (N = 102) and convenience sampling approach limit the generalizability of the findings beyond the participating university students. The sample was drawn from a university population and should not be considered representative of clinical populations or individuals receiving treatment for anxiety or other mental health conditions. A relatively substantial proportion of participants were from data analysis/computer science and cognitive science/communication-related fields, which may be associated with greater familiarity with digital technologies or AI. Prior chatbot use, technical familiarity with AI, and general attitudes toward technology were not systematically assessed, and their potential influence on participants’ responses could therefore not be evaluated.

Second, the questionnaire was administered in English to international university students. Participants were required to demonstrate a minimum IELTS score of 6 for university admission, providing an indication of English-language proficiency. However, comprehension of the questionnaire was not formally assessed through a separate language-validation or comprehension-testing procedure. Therefore, differences in language proficiency may have influenced individual responses.

Third, the study assessed participants’ perceptions and attitudes following exposure to a demonstration of the proposed framework rather than therapeutic effectiveness or clinical outcomes. Participants did not directly interact with the AI avatar; instead, they observed a brief live demonstration conducted either individually or in small groups. The demonstrations did not follow a standardized conversational script, and the immediate evaluation may have introduced novelty or presentation effects. Accordingly, the findings should not be interpreted as evidence of direct user experience, usability, feasibility, therapeutic effectiveness, or clinical utility.

Fourth, the questionnaire was developed specifically for this exploratory study and was not a previously validated psychometric instrument. Validated measures of anxiety symptoms, psychological distress, therapeutic alliance, usability, or clinical outcomes were not administered. The seven Likert-scale items were analyzed individually rather than as a validated composite scale. The findings should therefore be interpreted as exploratory information about participants’ reported perceptions rather than as evidence based on validated psychometric measures.

Fifth, the study did not include a comparison group or alternative experimental condition, and its cross-sectional design did not permit assessment of changes in perceptions or willingness to use AI-assisted support over time. Self-reported willingness to use AI-assisted support should therefore not be interpreted as evidence of actual adoption, sustained engagement, or real-world use.

Sixth, clinical supervision, safety outcomes, and crisis-management procedures were not evaluated in practice. Although clinician-informed principles were incorporated into the design of the proposed framework, no clinician continuously supervised participants’ exposure to the system or reviewed AI-generated responses during the study. The study therefore cannot establish the adequacy of the framework’s safety mechanisms or human-oversight procedures in real-world mental health settings.

Finally, several questionnaire items referred to an “AI-powered therapist.” This terminology may have influenced participants’ expectations regarding the role and capabilities of AI-assisted systems. In addition, the open-ended responses were reviewed descriptively without a formal qualitative coding framework, independent coding, or inter-rater reliability assessment. The recurring topics should therefore be interpreted as descriptive observations rather than as findings from a formal qualitative analysis.

Taken together, these limitations restrict the extent to which the findings can be generalized or used to support claims about therapeutic effectiveness, clinical utility, safety, or real-world implementation. The study should therefore be understood as a preliminary exploratory assessment of university students’ perceptions following exposure to a demonstration of the proposed AI-assisted framework. Future research should evaluate the framework through direct user interaction, validated outcome measures, appropriate comparison conditions, clinically relevant populations, longitudinal designs, and systematic assessment of safety and human oversight.

## 6. Conclusions

This study presented a hybrid AI-assisted framework for potential anxiety-related mental health support, integrating an embodied conversational avatar with clinician-informed dialogue strategies. An exploratory survey of 102 university students examined participants’ perceptions and attitudes toward AI-assisted mental health support following exposure to a live demonstration of the proposed framework. The findings showed that a proportion of participants perceived AI-assisted support as potentially useful and accessible, while reservations remained regarding emotional understanding, privacy, reliability, and the continued preference for human interaction.

These findings provide preliminary information about university students’ perceptions following exposure to a demonstration of the proposed framework. They should not be interpreted as evidence of therapeutic effectiveness, anxiety symptom reduction, clinical utility, or clinical safety. The proposed framework is intended as a potential complementary support tool within future human–AI mental health care models rather than as an autonomous replacement for qualified mental health professionals.

The findings highlight the importance of further evaluating AI-assisted mental health frameworks through direct user interaction, validated psychological and clinical outcome measures, appropriate comparison conditions, and clinically relevant populations. Future development should also incorporate clearly defined human oversight, safety and crisis-management procedures, privacy protections, and systematic evaluation of the reliability and appropriateness of AI-generated responses. Longitudinal research will be needed to determine whether perceptions and engagement are maintained over time and whether such frameworks can contribute meaningfully to future mental health applications.

## Figures and Tables

**Table 1:** Participant demographics.

Characteristic	Category	General Survey Cohort (*N* = 102)
Age, mean (SD)		26.0 (4.3)
Age range		19–38
**Gender, n (%)**		
	Female	52 (51.0%)
	Male	49 (48.0%)
	Non-binary	1 (1.0%)
**Prior therapy experience, n (%)**		
	Previous experience	48 (47.1%)
	No previous experience	36 (35.3%)
	Current experience	18 (17.6%)
**Primary field of study, n (%)**		
	Data analysis/computer science	38 (37.3%)
	Cognitive science/communication	20 (19.6%)
	Business/management	4 (3.9%)
	Other fields	40 (39.2%)

**Table 2:** Participants’ attitudes toward AI-assisted mental health systems (*N* = 102).

Item	Strongly Disagree, n (%)	Disagree, n (%)	Neither Agree nor Disagree, n (%)	Agree, n (%)	Strongly Agree, n (%)	Agree/Strongly Agree, n (%)	Median	IQR
Would feel comfortable talking to AI	4 (4.0%)	12 (11.9%)	26 (25.7%)	44 (43.6%)	15 (14.9%)	59 (58.4%)	4	1
AI could understand my emotions	12 (11.9%)	28 (27.7%)	37 (36.6%)	15 (14.9%)	9 (8.9%)	24 (23.8%)	3	1
Prefer real person over AI	4 (4.0%)	7 (7.0%)	34 (34.0%)	41 (41.0%)	14 (14.0%)	55 (55.0%)	4	1
AI could be useful for support	2 (2.0%)	10 (10.0%)	29 (29.0%)	44 (44.0%)	15 (15.0%)	59 (59.0%)	4	1
Uncomfortable sharing info with AI	10 (9.9%)	36 (35.6%)	31 (30.7%)	19 (18.8%)	5 (5.0%)	24 (23.8%)	3	1
Would use AI therapy if free	2 (2.0%)	10 (9.9%)	29 (28.7%)	36 (35.6%)	24 (23.8%)	60 (59.4%)	4	1
AI could help some people	3 (3.0%)	4 (4.0%)	17 (16.8%)	51 (50.5%)	26 (25.7%)	77 (76.2%)	4	1

Note. The overall survey sample included 102 participants. Percentages are based on valid item-level responses. Valid denominators varied across items because some participants did not provide a response to every item. Missing responses were excluded from the denominator for the corresponding item and were not imputed. Median and IQR were calculated based on valid responses for each individual Likert-scale item.

## Data Availability

The datasets generated during and/or analyzed during the current study are available from the corresponding author on reasonable request.

## Supplementary Materials

The following supporting information can be downloaded at: https://www.mdpi.com/article/10.3390/bs16101781/s1, Supplementary Questionnaire: Questionnaire Used to Assess University Students’ Perceptions of AI-Assisted Mental Health Support.

## Abbreviations

The following abbreviations are used in this manuscript:

AI	Artificial Intelligence
LLMs	Large Language Models
CBT	Cognitive Behavioral Therapy
NLP	Natural Language Processing

## References

[B1-behavsci-16-01781] Adindu K. N., Akubue N. E., Jude N. O., Onakoya A., Chukwunonye C., Odion O., Okengwu C. G., Nwudele U., Osita-Obasi P. Z., Ezike A., Bello I., Olenloa E., Eruteya O. O., Oyewole S. A. (2025). Artificial intelligence and digital therapy for adolescent mental health in the UK: Opportunities, barriers and ethical consideration. International Neuropsychiatric Disease Journal.

[B2-behavsci-16-01781] Ayoola A. A., Adeoye O. F., Ayoola A., Joy A. (2025). Telepsychiatry and digital mental health interventions: The future of psychiatric nursing. International Journal of Pharma Growth Research Review.

[B3-behavsci-16-01781] Békés V., Aafjes-van Doorn K. (2026). The most vulnerable are prone to use AI therapists: The role of attachment, epistemic trust, and mental health symptoms in acceptance of digital mental health interventions. Psychotherapy Research.

[B4-behavsci-16-01781] Daz 3D (2026). Introducing genesis 9.

[B5-behavsci-16-01781] He Y., Yang L., Qian C., Li T., Su Z., Zhang Q., Hou X. (2023). Conversational agent interventions for mental health problems: Systematic review and meta-analysis of randomized controlled trials. Journal of Medical Internet Research.

[B6-behavsci-16-01781] Kiuchi K., Otsu K., Hayashi Y. (2024). Psychological insights into the research and practice of embodied conversational agents, chatbots and social assistive robots: A systematic meta-review. Behaviour & Information Technology.

[B7-behavsci-16-01781] Li H., Zhang R., Lee Y. C., Kraut R. E., Mohr D. C. (2023). Systematic review and meta-analysis of AI-based conversational agents for promoting mental health and well-being. NPJ Digital Medicine.

[B8-behavsci-16-01781] Palmer A., Schwan D. (2025). Digital mental health tools and AI therapy chatbots: A balanced approach to regulation. Hastings Center Report.

[B9-behavsci-16-01781] Patel V., Saxena S., Lund C., Thornicroft G., Baingana F., Bolton P., Chisholm D., Collins P. Y., Cooper J. L., Eaton J., Herrman H., Herzallah M. M., Huang Y., Jordans M. J. D., Kleinman A., Medina-Mora M. E., Morgan E., Niaz U., Omigbodun O., Unützer J. (2018). The Lancet Commission on global mental health and sustainable development. The Lancet.

[B10-behavsci-16-01781] Pinto G. B. D., De Sousa J. P. R., Rosa R. L., Rodríguez D. Z. (2025). Embodied multimodal chatbot for mental health support in web-based 3D environments. 2025 International Conference on Software, Telecommunications and Computer Networks (SoftCOM).

[B11-behavsci-16-01781] Provoost S., Lau H. M., Ruwaard J., Riper H. (2017). Embodied conversational agents in clinical psychology: A scoping review. Journal of Medical Internet Research.

[B12-behavsci-16-01781] Radford A., Kim J. W., Xu T., Brockman G., McLeavey C., Sutskever I. (2023). Robust speech recognition via large-scale weak supervision. Proceedings of the 40th international conference on machine learning.

[B13-behavsci-16-01781] Rehm I. C., Foenander E., Wallace K., Abbott J. A. M., Kyrios M., Thomas N. (2016). What role can avatars play in e-mental health interventions? Exploring new models of client–therapist interaction. Frontiers in Psychiatry.

[B14-behavsci-16-01781] Rowan K., McAlpine D. D., Blewett L. A. (2013). Access and cost barriers to mental health care, by insurance status, 1999–2010. Health Affairs.

[B15-behavsci-16-01781] Shidara K., Tanaka H., Adachi H., Kanayama D., Kudo T., Nakamura S. (2024). Adapting the number of questions based on detected psychological distress for cognitive behavioral therapy with an embodied conversational agent: Comparative study. JMIR Formative Research.

[B16-behavsci-16-01781] Unity Technologies (n.d.). Unity 6: Download the latest release of Unity 6.

[B17-behavsci-16-01781] van Lotringen M. C., Jeken L., Westerhof G. J., ten Klooster P. M., Kelders S. M., Noordzij M. L. (2021). Responsible relations: A systematic scoping review of the therapeutic alliance in text-based digital psychotherapy. Frontiers in Digital Health.

[B18-behavsci-16-01781] Zhang Q., Zhang R., Xiong Y., Sui Y., Tong C., Lin F.-H. (2025). Generative AI mental health chatbots as therapeutic tools: Systematic review and meta-analysis of their role in reducing mental health issues. Journal of Medical Internet Research.

