# Supplementary material for: A Hybrid AI-Assisted Framework for Anxiety Support: System Description and Exploratory Findings Among University Students
Source: Behav Sci (Basel). 2026 Sep 30;16(10):1781. doi: 10.3390/bs16101781 (PMC13701661; doi:10.3390/bs16101781)
Supplement: Supplementary file 1 [file behavsci-16-01781-s001.zip › behavsci-4457315-supplementary.pdf]

COSPECS Department
University of Messina
Via Concezione 6-8, 98121 Messina

Informed Consent Form for Participation in the Study

RESEARCH TITLE: "Study of methodologies and psychological assessment aimed at the application of Artificial Intelligence and augmented/virtual reality systems to therapy and cognitive training and assistance".

NAME AND SURNAME AND TITLE OF THE MEMBERS OF THE RESEARCH GROUP:

Federica Iacopino - Psychologist-Psychotherapist
Amelia Gangemi - Full Professor
Giorgio Grasso - Associate Professor

INSTRUCTIONS: Before freely deciding whether you wish to participate in this study, **PLEASE READ THIS INFORMED CONSENT FORM CAREFULLY** and ask the person responsible for the research any questions you consider appropriate so that you are fully informed about the aims, procedures for conducting the experiment, and possible inconveniences involved. Before participating in the experimental session, we ask you to read this information sheet and any attached documents
whose purpose is to determine whether there are any reasons why your profile does not correspond to that required for the study in question. Your participation is completely voluntary. You may withdraw at any time without giving any explanation and request that the data collected be permanently deleted.

PURPOSE OF THE STUDY:
This research aims to evaluate the effectiveness of an intervention involving an AI-powered psychologist avatar in supporting students who suffer from performance anxiety and perceive high levels of academic stress.
The study will be conducted with students of the University of Messina and will assess whether the avatar can help manage anxiety symptoms, better recognize one's own emotions, and use practical strategies to cope with anxiety, including when it occurs.
The results will help us understand whether this type of technology may also be useful in other psychological contexts.

WHAT DOES THIS STUDY INVOLVE:

The study involves students of the University of Messina who responded to a questionnaire to collect information on age, gender, daily habits, recent stressful events, and anxiety symptoms (frequency, duration, intensity).
Based on the results, 20 students presenting moderate-level anxiety symptoms, which may nevertheless cause discomfort in daily life, will be selected. People with more serious psychological problems, who use drugs or medications, or who are already undergoing psychotherapy will not participate.
The selected participants will complete some psychological tests to assess their ability to recognize and manage emotions, anxiety sensitivity, anxiety symptoms, and perceived stress.
The program consists of 12 individual sessions with a psychologist, once a week, lasting approximately 50 minutes. The sessions are based on cognitive-behavioral therapy (CBT) techniques, useful for addressing negative thoughts, learning to manage anxiety, facing fears, and preventing relapse.
Each participant will also receive homework assignments and informational material to learn more about the disorder and use some strategies in daily life.

RISKS, DISCOMFORTS AND SIDE EFFECTS:

No risks

WHAT ARE THE POSSIBLE BENEFITS?
This study focuses on evaluating the effectiveness of an avatar specialized in managing anxiety in an academic setting. The aim is to provide immediate relief and long-term coping strategies.

WHAT WILL HAPPEN TO THE RESULTS OF THE STUDY?
The information obtained from these experiments may be published in scientific journals, but your name will not appear in any public document, nor will the results be published in a form that allows you to be identified.

WHERE ARE THE STUDY DATA STORED?
The data collected will be stored anonymously on the laboratory's and/or researcher's hard drive for a maximum of 7 years and will then be deleted.

RIGHT TO REFUSE OR WITHDRAW:
You may refuse to participate and/or withdraw from the study at any time without providing an explanation. Withdrawal during the study does not affect your right to compensation, if applicable. In addition, you may request that the data collected not be used for the purposes of this research. In that case, the available data will be permanently deleted.

QUESTIONS:
We are pleased to have the opportunity to answer any questions you may have about any aspect of this study or your participation in it. You may contact Dr. Federica Iacopino (email address: fecopino@gmail.com telephone number: 3711278771). Please refer to these same contacts if you need assistance or have questions or concerns after participating in the experiment.fecopino@gmail.com

WHAT HAPPENS IF I HAVE A COMPLAINT?
This research study has been approved by the Ethics Committee of the Department of Cognitive, Psychological, Pedagogical and Cultural Studies of the University of Messina. In case of complaints concerning the conduct of the research, you may contact Prof. Carmelo Mario Vicario, Chair of the Ethics Committee.

OTHER USEFUL INFORMATION:
Confidentiality. The data collected will be processed in accordance with privacy laws and in compliance with Legislative Decree No. 196 of 30 June 2003, "Code regarding the protection of personal data," guaranteeing the anonymity of participants.

Consent for participation in the study

Project title "Study of methodologies and psychological assessment aimed at the application of Artificial Intelligence and augmented/virtual reality systems to therapy and cognitive training and assistance".

Researcher: Federica Iacopino

I, the undersigned ____

Date of birth __________________________

Declares:

- to have carefully read and understood the explanations relating to this study and the entire experimental procedure;
- to have been informed about the purposes and objectives of the research in question; - to have had the opportunity to ask questions about any aspect of the experimental procedure and to have received satisfactory answers;
- to be aware of the discomforts of the experiment;
- to have received satisfactory assurances regarding the confidentiality of the information obtained from the examination of myself;
- to be aware that I may withdraw at any stage of the study;
- to have freely given consent to participate in this study;

Therefore, declares their willingness to participate

Date and place____________________________________

Signature of participant   Signature of researcher

__________________________   _____________________________
